# 2,4-Difluoro­phenyl­boronic acid

**DOI:** 10.1107/S1600536808040646

**Published:** 2008-12-10

**Authors:** Patricia Rodríguez-Cuamatzi, Hugo Tlahuext, Herbert Höpfl

**Affiliations:** a Universidad Politécnica de Tlaxcala, Carretera Federal Tlaxcala-Puebla Km 9.5, Tepeyanco, Tlaxcala, Mexico; b Centro de Investigaciones Químicas, Universidad Autónoma del Estado de Morelos. Av. Universidad 1001 Col., Chamilpa, CP 62209, Cuernavaca Mor., Mexico

## Abstract

The mol­ecular structure of the title compound, C_6_H_5_BF_2_O_2_, is essentially planar (mean deviation = 0.019 Å), indicating electronic delocalization between the dihydroxy­boryl group and the aromatic ring. In the crystal structure, inversion dimers linked by two O—H⋯O hydrogen bonds arise. An intra­molecular O—H⋯F hydrogen bond reinforces the conformation and the same H atom is also involved in an inter­molecular O—H⋯F link, leading to mol­ecular sheets in the crystal.

## Related literature

For general backround to boronic acids, see: Hall (2005 ▶); Höpfl (2002 ▶); Fujita *et al.* (2008 ▶); Soloway *et al.* (1998 ▶). For hydrogen-bond motifs, see: Bernstein *et al.* (1995 ▶); Desiraju (2002 ▶). For related structures, see: Wu *et al.* (2006 ▶); Bradley *et al.* (1996 ▶); Horton *et al.* (2004 ▶). For crystal engineering, see: Fournier *et al.* (2003 ▶); Rodríguez-Cuamatzi *et al.* (2004 ▶, 2005 ▶).
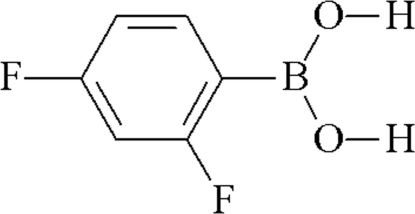


## Experimental

### 

#### Crystal data

C_6_H_5_BF_2_O_2_
*M* *_r_* = 157.91
Monoclinic, 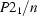

*a* = 3.7617 (11) Å
*b* = 12.347 (4) Å
*c* = 14.620 (4) Å
β = 95.450 (5)°
*V* = 676.0 (3) Å^3^
*Z* = 4
Mo *K*α radiation
μ = 0.15 mm^−1^
*T* = 293 (2) K
0.37 × 0.35 × 0.22 mm

#### Data collection

Bruker SMART APEX CCD area-detector diffractometer
Absorption correction: multi-scan (*SADABS*; Sheldrick, 1996 ▶) *T*_min_ = 0.947, *T*_max_ = 0.968
3196 measured reflections
1190 independent reflections
1012 reflections with *I* > 2σ(*I*)
*R*_int_ = 0.028

#### Refinement

*R*[*F* ^2^ > 2σ(*F* ^2^)] = 0.056
*wR*(*F* ^2^) = 0.127
*S* = 1.15
1190 reflections
106 parameters
2 restraints
H atoms treated by a mixture of independent and constrained refinement
Δρ_max_ = 0.14 e Å^−3^
Δρ_min_ = −0.18 e Å^−3^

### 

Data collection: *SMART* (Bruker, 2000 ▶); cell refinement: *SAINT-Plus-NT* (Bruker, 2001 ▶); data reduction: *SAINT-Plus-NT*; program(s) used to solve structure: *SHELXTL-NT* (Sheldrick, 2008 ▶); program(s) used to refine structure: *SHELXTL-NT*; molecular graphics: *CAMERON* (Watkin *et al.*, 1996 ▶); software used to prepare material for publication: *PLATON* (Spek, 2003 ▶) and *publCIF* (Westrip, 2009 ▶).

## Supplementary Material

Crystal structure: contains datablocks I, global. DOI: 10.1107/S1600536808040646/hb2865sup1.cif

Structure factors: contains datablocks I. DOI: 10.1107/S1600536808040646/hb2865Isup2.hkl

Additional supplementary materials:  crystallographic information; 3D view; checkCIF report

## Figures and Tables

**Table 1:** Hydrogen-bond geometry (Å, °)

*D*—H⋯*A*	*D*—H	H⋯*A*	*D*⋯*A*	*D*—H⋯*A*
O1—H1⋯F1	0.841 (15)	2.16 (3)	2.799 (3)	133 (2)
O1—H1⋯F2^i^	0.841 (15)	2.39 (2)	3.086 (3)	140 (3)
O2—H2⋯O1^ii^	0.841 (19)	1.97 (2)	2.809 (3)	174 (3)

Symmetry codes: (i) 
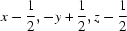
; (ii) 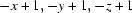.

**Fig. 1.**
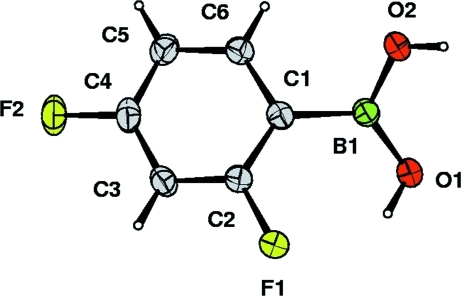
The molecular structure of (I) with displacement ellipsoids drawn at the 30% probability level and H atoms shown as small spheres of arbitrary radius.

**Fig. 2.**
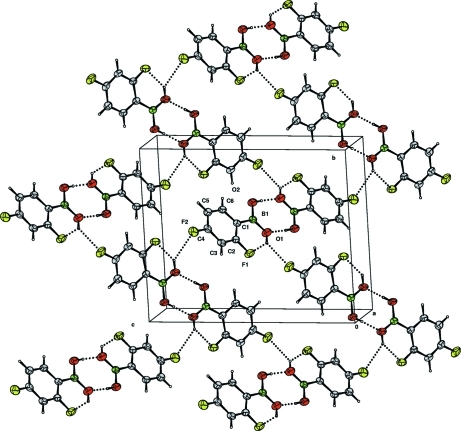
View of the packing arrangement of the two-dimensional network of (I)(I).

## supplementary crystallographic information

### Comment

Boronic acids, RB(OH)_2_ with *R* = alkyl and aryl, have applications in organic synthesis (Hall, 2005), host–guest chemistry (Höpfl, 2002), the molecular recognition of biochemically active molecules (Fujita *et al.*, 2008) and in medicine as antibiotics, inhibitors and for the treatment of tumors (Soloway *et al.*, 1998). Similar to carboxylic acids they are capable to form hydrogen-bonded dimeric units and, therefore, boronic acids have been used recently as new building blocks in crystal engineering (Fournier *et al.*, 2003; Rodríguez-Cuamatzi *et al.*, 2004; Rodríguez-Cuamatzi *et al.*, 2005). Previously, the structures of 3-fluorophenylboronic acid (Wu *et al.*, 2006), 2,6-difluoroboronic acid (Bradley *et al.*, 1996) and pentafluoroboronic acid (Horton *et al.*, 2004) had been reported. We now present the crystal structure of (I).

The molecular structure is essentially planar, O1—B1—C1—C2 = 4.4 (4)°, indicating that there is a π···π interaction between the dihydroxyboryl group and the aromatic ring, to which it is attached (Fig. 1). This interaction is also evidenced by the B—C bond length of 1.566 (3) Å, which is significantly shorter than that observed in boronates containing tetra-coordinate boron atoms (Höpfl, 2002). The crystal structure is stabilized by strong O2—H2···O1 hydrogen-bonding interactions, forming *R*_2_^2^(8) motifs (Bernstein *et al.*, 1995), as well as, O1—H1···F1 and O1—H1···F2 bifurcated hydrogen bonds (Fig. 2; Table 1) (Desiraju, 2002). Due to these interactions each boronic acid homodimer is linked to two neighboring homodimeric units, thus creating a two-dimensional hydrogen-bonded network, in which fluorine is therefore an essential structural component.

### Experimental

2,4-Difluorophenylboronic acid was purchased from Aldrich and crystallized from water to yield colourless blocks of (I).

### Refinement

The aromatic H atoms were positioned geometrically (C—H = 0.93Å) and refined as riding with *U*_iso_(H) = 1.2*U*_eq_(C). The O—H hydrogen atoms were localized in a difference map and their coordinates were refined with O—H = 0.84+/0.01Å and *U*_iso_(H) = 1.5 *U*_eq_(O).

### Crystal data

**Table:**

C_6_H_5_BF_2_O_2_	*F*(000) = 320
*M**_r_* = 157.91	*D*_x_ = 1.552 Mg m^−^^3^
Monoclinic, *P*2_1_/*n*	Melting point = 521–522 K
Hall symbol: -P 2yn	Mo *K*α radiation, λ = 0.71073 Å
*a* = 3.7617 (11) Å	Cell parameters from 1052 reflections
*b* = 12.347 (4) Å	θ = 2.3–26.2°
*c* = 14.620 (4) Å	µ = 0.15 mm^−^^1^
β = 95.450 (5)°	*T* = 293 K
*V* = 676.0 (3) Å^3^	Block, colorless
*Z* = 4	0.37 × 0.35 × 0.22 mm

### Data collection

**Table:**

Bruker SMART APEX CCD area-detector diffractometer	1190 independent reflections
Radiation source: fine-focus sealed tube	1012 reflections with *I* > 2σ(*I*)
graphite	*R*_int_ = 0.028
Detector resolution: 8.3 pixels mm^-1^	θ_max_ = 25.0°, θ_min_ = 2.2°
φ and ω scans	*h* = −3→4
Absorption correction: multi-scan (*SADABS*; Sheldrick, 1996)	*k* = −14→12
*T*_min_ = 0.947, *T*_max_ = 0.968	*l* = −17→17
3196 measured reflections	

### Refinement

**Table:**

Refinement on *F*^2^	Primary atom site location: structure-invariant direct methods
Least-squares matrix: full	Secondary atom site location: difference Fourier map
*R*[*F*^2^ > 2σ(*F*^2^)] = 0.056	Hydrogen site location: inferred from neighbouring sites
*wR*(*F*^2^) = 0.127	H atoms treated by a mixture of independent and constrained refinement
*S* = 1.15	*w* = 1/[σ^2^(*F*_o_^2^) + (0.0442*P*)^2^ + 0.2673*P*] where *P* = (*F*_o_^2^ + 2*F*_c_^2^)/3
1190 reflections	(Δ/σ)_max_ < 0.001
106 parameters	Δρ_max_ = 0.14 e Å^−^^3^
2 restraints	Δρ_min_ = −0.18 e Å^−^^3^

### Special details

**Table:**

Geometry. All e.s.d.'s (except the e.s.d. in the dihedral angle between two l.s. planes) are estimated using the full covariance matrix. The cell e.s.d.'s are taken into account individually in the estimation of e.s.d.'s in distances, angles and torsion angles; correlations between e.s.d.'s in cell parameters are only used when they are defined by crystal symmetry. An approximate (isotropic) treatment of cell e.s.d.'s is used for estimating e.s.d.'s involving l.s. planes.
Refinement. Refinement of *F*^2^ against ALL reflections. The weighted *R*-factor *wR* and goodness of fit *S* are based on *F*^2^, conventional *R*-factors *R* are based on *F*, with *F* set to zero for negative *F*^2^. The threshold expression of *F*^2^ > σ(*F*^2^) is used only for calculating *R*-factors(gt) *etc*. and is not relevant to the choice of reflections for refinement. *R*-factors based on *F*^2^ are statistically about twice as large as those based on *F*, and *R*- factors based on ALL data will be even larger.

### Fractional atomic coordinates and isotropic or equivalent isotropic displacement parameters (Å^2^)

**Table:**

	*x*	*y*	*z*	*U*_iso_*/*U*_eq_	
B1	0.7704 (8)	0.4548 (2)	0.62567 (18)	0.0452 (7)	
O1	0.6825 (6)	0.38623 (15)	0.55419 (13)	0.0695 (6)	
H1	0.748 (9)	0.3215 (9)	0.562 (2)	0.104*	
O2	0.6880 (6)	0.55977 (14)	0.61557 (12)	0.0630 (6)	
H2	0.593 (8)	0.577 (3)	0.5632 (10)	0.094*	
F1	1.0385 (5)	0.23728 (11)	0.67473 (11)	0.0768 (6)	
F2	1.4329 (5)	0.33016 (14)	0.97634 (10)	0.0822 (6)	
C1	0.9591 (6)	0.41789 (18)	0.72069 (15)	0.0424 (6)	
C2	1.0796 (7)	0.31430 (18)	0.74175 (16)	0.0470 (6)	
C3	1.2380 (7)	0.2819 (2)	0.82553 (17)	0.0539 (7)	
H3	1.3138	0.2109	0.8363	0.065*	
C4	1.2785 (7)	0.3593 (2)	0.89223 (17)	0.0547 (7)	
C5	1.1696 (8)	0.4640 (2)	0.87828 (17)	0.0586 (7)	
H5	1.2013	0.5150	0.9251	0.070*	
C6	1.0119 (7)	0.49169 (19)	0.79282 (16)	0.0498 (6)	
H6	0.9371	0.5628	0.7827	0.060*	

### Atomic displacement parameters (Å^2^)

**Table:**

	*U*^11^	*U*^22^	*U*^33^	*U*^12^	*U*^13^	*U*^23^
B1	0.0451 (16)	0.0425 (15)	0.0471 (16)	−0.0009 (12)	−0.0005 (12)	0.0033 (12)
O1	0.1000 (17)	0.0484 (11)	0.0540 (11)	0.0158 (10)	−0.0241 (10)	−0.0009 (9)
O2	0.0850 (15)	0.0441 (10)	0.0552 (11)	0.0087 (9)	−0.0172 (10)	0.0048 (8)
F1	0.1192 (15)	0.0456 (9)	0.0602 (10)	0.0151 (9)	−0.0187 (9)	−0.0046 (7)
F2	0.1070 (14)	0.0804 (12)	0.0527 (10)	−0.0120 (10)	−0.0262 (9)	0.0181 (8)
C1	0.0383 (13)	0.0412 (13)	0.0473 (13)	−0.0039 (10)	0.0019 (10)	0.0048 (10)
C2	0.0523 (16)	0.0410 (13)	0.0467 (13)	−0.0017 (11)	0.0001 (11)	0.0008 (10)
C3	0.0584 (17)	0.0449 (14)	0.0564 (15)	0.0002 (12)	−0.0053 (13)	0.0124 (12)
C4	0.0579 (17)	0.0613 (17)	0.0426 (13)	−0.0099 (13)	−0.0075 (12)	0.0135 (12)
C5	0.0696 (19)	0.0552 (16)	0.0489 (14)	−0.0109 (13)	−0.0058 (13)	−0.0020 (12)
C6	0.0559 (16)	0.0399 (13)	0.0522 (14)	−0.0011 (11)	−0.0011 (12)	0.0029 (11)

### Geometric parameters (Å, °)

**Table:**

B1—O2	1.338 (3)	C1—C6	1.394 (3)
B1—O1	1.361 (3)	C2—C3	1.370 (3)
B1—C1	1.566 (3)	C3—C4	1.363 (4)
O1—H1	0.841 (15)	C3—H3	0.93
O2—H2	0.841 (15)	C4—C5	1.366 (4)
F1—C2	1.364 (3)	C5—C6	1.374 (3)
F2—C4	1.358 (3)	C5—H5	0.93
C1—C2	1.382 (3)	C6—H6	0.93
			
O2—B1—O1	118.7 (2)	C4—C3—H3	121.8
O2—B1—C1	117.4 (2)	C2—C3—H3	121.8
O1—B1—C1	123.8 (2)	F2—C4—C3	118.1 (2)
B1—O1—H1	116 (2)	F2—C4—C5	118.8 (2)
B1—O2—H2	115 (2)	C3—C4—C5	123.0 (2)
C2—C1—C6	114.6 (2)	C4—C5—C6	117.9 (2)
C2—C1—B1	125.3 (2)	C4—C5—H5	121.0
C6—C1—B1	120.1 (2)	C6—C5—H5	121.0
F1—C2—C3	116.7 (2)	C5—C6—C1	122.9 (2)
F1—C2—C1	118.2 (2)	C5—C6—H6	118.5
C3—C2—C1	125.1 (2)	C1—C6—H6	118.5
C4—C3—C2	116.4 (2)		
			
O2—B1—C1—C2	−176.5 (2)	C1—C2—C3—C4	−0.3 (4)
O1—B1—C1—C2	4.5 (4)	C2—C3—C4—F2	179.7 (2)
O2—B1—C1—C6	4.6 (4)	C2—C3—C4—C5	0.0 (4)
O1—B1—C1—C6	−174.5 (2)	F2—C4—C5—C6	−179.6 (2)
C6—C1—C2—F1	−179.9 (2)	C3—C4—C5—C6	0.1 (4)
B1—C1—C2—F1	1.1 (4)	C4—C5—C6—C1	0.0 (4)
C6—C1—C2—C3	0.4 (4)	C2—C1—C6—C5	−0.3 (4)
B1—C1—C2—C3	−178.6 (2)	B1—C1—C6—C5	178.8 (2)
F1—C2—C3—C4	180.0 (2)		

### Hydrogen-bond geometry (Å, °)

**Table:**

*D*—H···*A*	*D*—H	H···*A*	*D*···*A*	*D*—H···*A*
O1—H1···F1	0.84 (2)	2.16 (3)	2.799 (3)	133 (2)
O1—H1···F2^i^	0.84 (2)	2.39 (2)	3.086 (3)	140 (3)
O2—H2···O1^ii^	0.84 (2)	1.97 (2)	2.809 (3)	174 (3)

Symmetry codes: (i) *x*−1/2, −*y*+1/2, *z*−1/2; (ii) −*x*+1, −*y*+1, −*z*+1.
